# Prevalence of celiac disease in primary care: the need for its own code

**DOI:** 10.1186/s12913-019-4407-4

**Published:** 2019-08-16

**Authors:** Ricardo Fueyo-Díaz, Rosa Magallón-Botaya, Barbara Masluk, Guillermo Palacios-Navarro, Angela Asensio-Martínez, Santiago Gascón-Santos, Bárbara Olivan-Blázquez, Juan José Sebastián-Domingo

**Affiliations:** 1 Aragon Institute of Health Sciences IACS, Zaragoza, Spain; 2 Department of Psychology and Sociology, University of Zaragoza, Zaragoza, Spain; 3 Department of Medicine, Psychiatry and Dermatology, University of Zaragoza, Zaragoza, Spain; 4 Institute of Health Research of Aragon (IIS), Zaragoza, Spain; 5 Department of Electronic Engineering and Communications, University of Zaragoza, Zaragoza, Spain; 6 Hospital Royo Villanova, Zaragoza, Spain

**Keywords:** Celiac disease, Prevalence, Diagnosis, Primary care, epidemiological research

## Abstract

**Background:**

Celiac disease (CD) is an autoimmune chronic enteropathy of the small intestine caused by exposure to gluten in genetically predisposed individuals. CD is not easy to diagnose due to its unspecific symptomatology, especially in adults, a diagnosed/undiagnosed ratio of 1:7 is estimated. CD does not have its own code in the International Classification of Primary Care (ICPC) but it is coded under code D99 “Disease digestive system, other”, which hinders diagnosis, intervention and research. The aim of this study is to investigate the prevalence of CD in Aragón, Spain, using the information available from Primary Care, as well as to discuss the difficulties involved in determining prevalence of CD from data collected at this level of medical intervention.

**Methods:**

We designed an epidemiological cross-sectional study and analysed 26,964 electronic clinical records from the Aragonese Health Service under code ICPC D99 collected up to December 31st, 2016. The clinical records were classified by their editable field “descriptor” according to their probability of being related to CD. Analyses of gender, age, age at diagnosis, province and health sector were carried out.

**Results:**

We found 4534 clinical records under 293 different descriptors with a high probability of referring to CD. Prevalence in Aragón was estimated to be 0.35% ranging from 0.24 to 0.81% with important differences among health sectors.

**Conclusions:**

The prevalence of 0.35% is a long way from the generally accepted 1% but within the usually considered ratio 1:7 of diagnosed:undiagnosed cases. Differences among sectors should be carefully analysed. Lacking its own ICPC code, diagnosis of CD in Primary Care Services is not included in a single category, but it is distributed under several descriptors, which makes it difficult to offer any firm diagnosis for treatment and hinders research. Finally, the high prevalence of CD justifies its own ICPC code and the need to withdraw CD from the generic D99 code “Disease digestive system other”.

## Background

According to the *Oslo definitions* [1]*,* celiac disease is a chronic small intestinal immune-mediated enteropathy triggered by exposure to dietary gluten in genetically predisposed people. This gluten is present in cereals such as wheat, barley, rye and, probably, in some types of oats [2]. This causes serious enteropathy of the small intestinal mucosa, which hinders correct nutrient uptake.

According to the European Society for Paediatric Gastroenterology Hepatology and Nutrition (ESPGHAN), the diagnosis of CD relies on gluten-dependent symptoms, CD-specific antibody levels, the presence of HLA-DQ2 and/or HLA-DQ8, and characteristic histological changes in the duodenal biopsy [3]. Currently, the only known treatment for CD is to follow a strict gluten-free diet for life.

CD is one of the most common chronic intestinal diseases [4]. Since CD is genetically mediated [1, 3], its prevalence depends on the frequency distribution of the HLA-DQ2 and HLA-DQ8 haplotypes among population [5]. Depending on the country, this distribution of HLA-DQ2 and DQ-8 reaches up to 40% [6] but the trigger factors for CD still remain unknown [7]. Previous studies report a prevalence for CD of 0.71% [8] in the USA and 0.1–2.8% in Europe [9], while a 1% prevalence is widely accepted [10]. Unfortunately there are few studies on prevalence of CD in Spain [11–15] and furthermore, differences among them are considerable, ranging from 0.3 to 1.4% [3]. No environmental or lifestyle factors that may explain these differences have been identified. Studies on prevalence indicate a 2.8:1 female/male ratio [16]. Some research suggests that this gender difference may be due to a later diagnosis in males [17] or to the fact that the non-classical forms of the disease may remain undiagnosed in men [18].

In recent decades, prevalence studies have shown that CD is a worldwide, frequent disease, which affects both children and adults [7, 10]. It is difficult to diagnose on account of the variety of symptoms it presents [1]. Only a small part of people affected by CD would show the classic, evident signs of the disease, while the majority would have the asymptomatic form. Thus, the variety of clinical symptoms of this illness hinders its diagnosis and would explain an underdiagnosis of 1:3 to 1:9 of diagnosed/undiagnosed cases [3, 18–20].

Traditionally, professionals have used different terms to refer to CD. Terms such as sprue, coeliac sprue, non-tropical sprue, idiopathic steatorrhea, gluten-sensitive enteropathy and gluten intolerance have been used as equivalents, but nowadays, their use is discouraged by the Oslo Group [1]. This group discourages the use of *gluten intolerance* and *gluten sensitivity,* which should be replaced by *celiac disease* or by *non-celiac gluten sensitivity*, respectively. Gluten sensitivity, therefore, would refer to those individuals with clinical manifestations triggered by gluten intake when CD has been excluded.

The International Classification of Primary Care is a classification method for Primary Care Services. It contributes to labelling the patient’s *reason for encounter* (RFE), to managing diagnosis, to designing primary health care interventions and to order data for research. It was developed by the WONCA International Classification Committee (WICC) and was first published in 1987. A revision with new criteria and definitions was published in 1998. It was accepted by the World Health Organization’s (WHO) Family of International Classifications. This classification was developed in response to the need of quality information on Primary Care.

From the early 2000s, the Aragonese Health Care Service launched the implementation of electronic clinical records in all its Primary Care centres, a process which was completed in 2011. Those records are coded according to this international classification.

The International Classification of Primary Care contains 17 chapters with Chapter D being for Digestive Diseases". Within this chapter, CD is coded under D99 “Disease digestive system other”, as there is no specific label for CD.

The autonomous community of Aragon is located in northeastern Spain. It is composed of three provinces, from north to south: Huesca, Zaragoza, and Teruel. It is divided into 8 health sectors with their own secondary health services and 127 health areas. Aragon has 1.3 million inhabitants. It comprises 33 counties.

Previous research based on data of 2011 estimated the prevalence of CD in Aragon at 0.15% (0.36% in Huesca, 0.12% in Zaragoza and 0.07% in Teruel) [21]. The increase of social awareness and the better knowledge of CD in recent years lead us to hypothesize a significant growth of the prevalence of CD in 2016 compared with 2011.

Therefore, the objectives of this investigation were: i) to study the quality of the information regarding CD in Primary Care available to General Practitioners. ii) to study the prevalence of CD in the community from the information in Primary Care.

## Methods

### Participants

The study population comprised patients in the Aragonese Health Services with code D99 (“Disease digestive system, other”) in the ICPC up to December 31, 2016. Data were requested from the Department of Health, with the following anonymized variables: patient code, gender, date of birth, descriptor, date of diagnosis, doctor’s code, area of residence, centre, zip code and nationality. These data were produced in the daily practice of general practitioners in their Primary Care centre.

### Classification process

26,965 clinical records of patients with an age range of 1 to 107 years old from 842 municipalities in Aragon were analysed.

Since all clinical records belonged to D99 “Disease digestive system, other”, records were classified using the database field named “descriptor”, in which family doctors can introduce their clinical impression.

The research team classified the cases into three groups on the basis of their descriptors and according to the patient’s likelihood of a suffering from CD: “*strong evidence group”* (with a descriptor making specific mention to a firm CD diagnosis or gluten intolerance); “*weak evidence group”* (with references to *gluten* or CD, but no firm diagnosis); and the rest of the clinical records were assigned to the “*no evidence group”.*

### Statistical analysis

Distribution by province, gender, age, date of diagnosis, age at diagnosis, medical centre, doctor and nationality was analysed. In order to compare the prevalence of CD in different periods, a two-sample test of proportions was carried out. Previously, data were standardised for gender and age and 0.05 was taken as being statistically significant throughout.

SPSS v.25 programme was used for descriptive statistical analysis and this study was approved by the Ethics Committee of Research of Aragon (CEICA), registered under number PI 14/0011.

## Results

The analysis of the 26,965 records showed 4534 (16,81%) *strong evidence CD* cases distributed in 293 different descriptors. There were other 1198 (4,44%) patients with *weak evidence* of suffering from the disease, distributed under 729 additional descriptors. The 134 cases under the descriptor “malabsorption syndrome” were assigned to this *weak evidence group* because their etiology may be different to CD. 8 cases described as “gluten sensitive” were expressly assigned to this second group. Finally, 21,233 were classified into the *no evidence group* (Table 1).

**Table 1:** Group classification by probability of suffering from CD

Group	n
D99 code	26,965
Strong evidence group	4534 in 293 descriptors
“NC celiac disease”, “celiac disease” and “Celiac. Disease”	3637 (80.22%)
“Gluten enteropathy”, “gluten enteropat. by” and others	897
Weak evidence group	1198 in 729 descriptors
No evidence group	21,233

In the *strong evidence group*, only 3637 (80,2%) were under the unmistakable categories of *NC celiac disease*, *celiac disease* and *Celiac, Disease*, while the remaining 897 came under headings, such as *“gluten enteropathy”*, *“gluten enteropat. by”* or other categories edited by family doctors and classified into the *strong evidence group* by this research team (Table 2).

**Table 2:** Examples of descriptors for the *strong evidence group*. Boldfaced terms are discouraged by Oslo Group

Rank	Descriptor	n	Percentage	Cumulative percentage
1.	Celiac Disease NC	1,972	43.5	43.5
2.	Celiac, Disease	1,539	33.9	77.4
**3.**	**Enteropathy by gluten**	368	8.1	85.6
4.	Gluten enteropat. by	141	3.1	88.7
5.	Coeliac Disease	126	2.8	91.4
**6.**	**Intolerance to gluten**	27	0.6	92
7.	Celiac	17	0.4	92.4
**8.**	**Gluten Intolerance**	15	0.3	92.7
9.	Adult Celiac Disease	6	0.1	92.9
10.	Coeliac	5	0.1	93
**11.**	**Intolerance to gluten and lactose**	5	0.1	93.1
12.	Others	313	6.9	100

Note: Authors have decided to literally translate the terms into English, as they appear to GPs in the clinical records, to show the abuse of slightly different terms when referring to Celiac Disease

Of the 4534 CD cases identified (71.8% female), the youngest patient was 2 years old and the oldest 102 (M: 38.97; SD: 20.85) (Table 3).

**Table 3:** Demographic characteristics in patients with CD (strong evidence group)

Characteristic	2016
Mean age (SD/Range)	38.97(20.85/2–102)
Female (%)	71.8
Mean age at diagnosis (SD/Range)	32.42 (21.05/0.5–98)
Mode (year of diagnosis)	2016
% cases/year (≤2010/2011/2012/2013/2014/2015/2016)	37.5/8.3/8.7/10.3/11/11.5/12.6
Nationalities	36

**Fig. 1:**
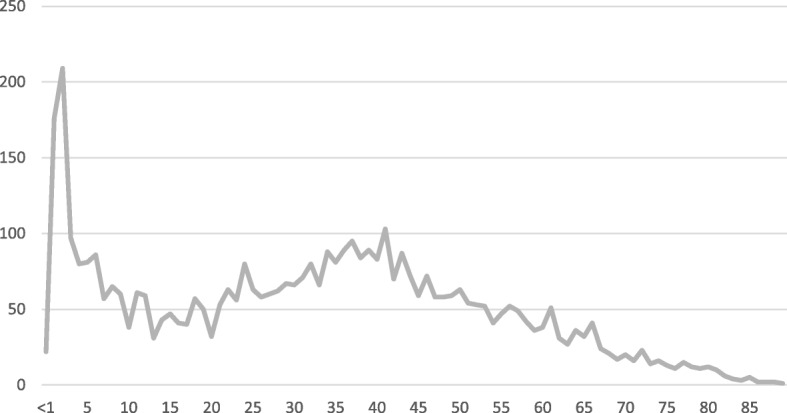
Age at diagnosis

Mean age at diagnosis was 32.42 years, with a standard deviation of 21.05, but with a range of just a few months to 98 years (Fig. 1).

**Fig. 2:**
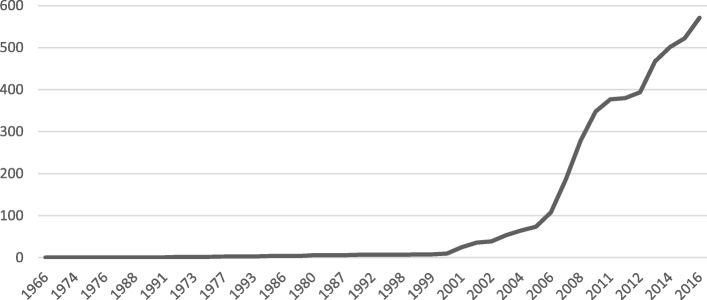
Year of diagnosis

The mode for year of diagnosis was 2016 with 571 cases. A significant increase in diagnoses is observed from 2000, reaching more than 500 cases per year in the last three years of the study (Fig. 2).

### Prevalence

Prevalence of CD was estimated in 0.35% in Aragon. Prevalence by province was 0.81% in Huesca, 0.25% in Zaragoza and 0.24% in Teruel. Regarding their nationality, 4362 (96.2%) were Spanish citizens and the rest were from 20 other different nationalities.

The analysis by health sectors is shown in Table 4. Only sector 4 approaches the expected prevalence of 1%, while most of the sectors are within the usual diagnosed/undiagnosed ratio of 1:7.

**Table 4:** Prevalence of CD by health sector with different secondary health services

Health Sector	Prevalence 2016
Sector 1	0.13%
Sector 2	0.44%
Sector 3	0.28%
Sector 4	1.16%
Sector 5	0.31%
Sector 6	0.26%
Sector 7	0.21%
Sector 8	0.34%

When we analyse the prevalence by age group, we see that it is similar for children and adolescents. However, as age increases, important differences appear among ratios, being Teruel the province with lower figures. Prevalence in Huesca shows an atypical evolution through age, with better figures between 30 and 59 years old (Table 5).

**Table 5:** Prevalence by age in Aragon and provinces

PREVALENCIA	0–14	15–29	30–44	45–59	60–74	75–89	≥90
ARAGÓN	0.38%	0.47%	0.87%	0.34%	0.27%	0.18%	0.12%
Huesca	0.48%	0.79%	1.06%	1.04%	0.79%	0.40%	0.23%
Teruel	0.50%	0.33%	0.21%	0.21%	0.16%	0.08%	0.04%
Zaragoza	0.34%	0.41%	0.25%	0.20%	0.17%	0.13%	0.11%

**Fig. 3:**
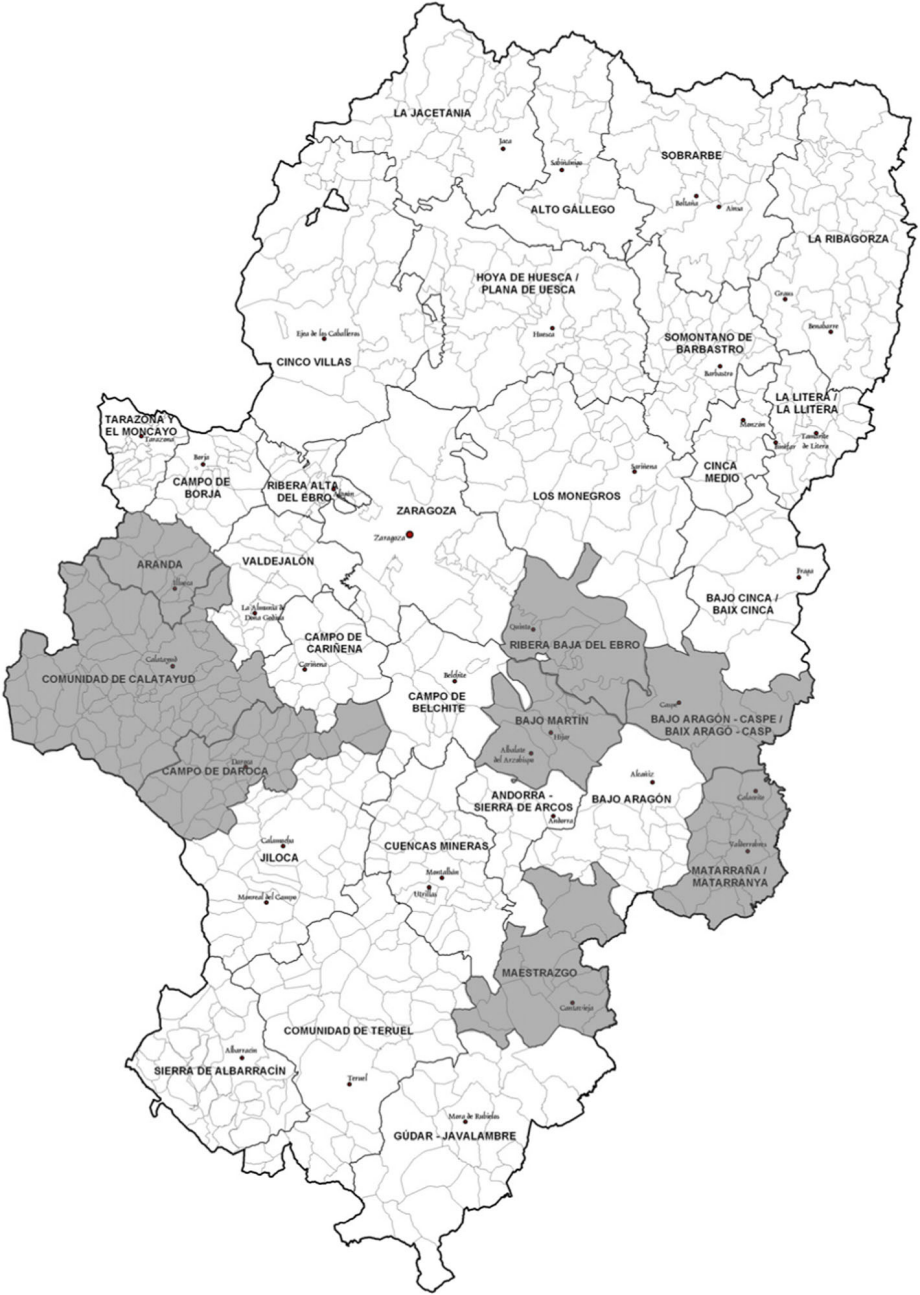
Prevalence of celiac disease in Aragon, Spain. Prevalence lower than 1:700 is shown in grey. Image modified (own work), via Wikimedia Commons, available at https://commons.wikimedia.org/wiki/File:Map_of_municipalities,_comarcas_and_provinces_of_Aragon.svg

An analysis by counties reveals important differences among them (Fig. 3).

Our study shows that the number of detected cases has duplicated in Aragon since 2011, in comparison with those found in the previous research [21] (Table 6).

**Table 6:** Evolution of CD prevalence in Aragon 2011–2016

	2011	2016
N patients with CD in descriptors	2.042 in 93 descriptors	4534 in 293 descriptors
Prevalence Aragón	0.15%	0.35%
Huesca	0.36%	0.81%
Zaragoza	0.12%	0.25%
Teruel	0.07%	0.24%
Age mean (SD/Range)	34.82 (22.18/1–92)	38.97(20.85/2–102)
% Female	71.48%	71.8
Age at diagnosis mean (SD/Range)	30.93 years, (22.54/< 1–88)	32.42 (21.05/0,5–98)
Nationalities	21	36

If we standardise the prevalence for gender and age (Table 7), and we compare both periods, all values are significant (*p* < 0.05) and therefore, we can conclude that prevalence of CD is greater in 2016 than in 2011, both in the whole of Aragon and in each province.

**Table 7:** Comparison of standardized prevalences between 2011 and 2016

	2011		2016		
	Crude Prevalence (%)	Standardised Prevalence (%) (^a^)	Crude Prevalence (%)	Standardised Prevalence (%) (^a^)	*p* value
Aragón	0.1512	0.1511	0.3464	0.3481	< 0.001**
Huesca	0.3585	0.3580	0.8120	0.8116	< 0.001**
Teruel	0.0668	0.0666	0.2383	0.2389	< 0.001**
Zaragoza	0.1154	0.1153	0.2535	0.2559	< 0.001**

^(a)^ Standardised prevalence for age and gender

^(**)^ Significant differences (*p* ≤ 0.05)

Note: we allow four decimal places to show differences

## Discussion

### Prevalence

This study shows that the number of detected cases has duplicated in Aragon since 2011 [21]. Although these results are promising, they are far from the generally accepted figure of 1% [9, 22]. Several factors may account for these results such as: a) the improvement of methods of diagnosis, b) stronger awareness among general practitioners when searching for CD in children and adults, c) greater patient awareness towards unspecific symptomatology, which will make them persevere to obtain a firm diagnosis.

According to our research, prevalence in Aragon stands at 0.35%. It should be noted that this remains far from the general accepted prevalence, but it falls within the underdiagnosis of 1:5–7 [16], showing a good detection capacity from the National Health Service, at least similar to nearby countries. The prevalence found in Huesca is three times higher than in Zaragoza and five times higher than in Teruel, which points to a lack of unified diagnostic criteria, methods or resources among health sectors. These differences are not so visible in children from 0 to 16 when they are under pediatric supervision, in comparison with older patients. This is probably due to the difficulty of diagnosing CD in adults, as they might remain asymptomatic or show non-classic forms of CD. Seronegative CD reaches up to 22% of all diagnosed cases [7], which shows that this technique is insufficient to be used in Primary Care as the only method to discard CD. As a result, these patients can be misdiagnosed with irritable bowel syndrome or other gastrointestinal diseases [22]. The mean age at diagnosis at 32 years, highlights that CD is not only a child’s disease, but one that can appear at any stage of life [23, 24]. Diagnosis is more frequent during the first and the fourth decade.

Differences among health sectors are important and it is interesting to note that sectors 2, 4 and 8 have been the most effective when detecting CD, both in 2011 and 2016, while sector 1 shows the lowest prevalence of all in both studies (Table 8).

**Table 8:** Comparative prevalence of CD among health sectors in 2011 and 2016

Ranking 2016	Health Sector	Prevalence 2016	Prevalence 2011	Ranking 2011
1	Sector 4	1.16%	0.59%	1
2	Sector 2	0.44%	0.14%	3
3	Sector 8	0.34%	0.17%	2
4	Sector 5	0.31%	0.09%	6
5	Sector 3	0.28%	0.05%	7
6	Sector 6	0.26%	0.12%	4
7	Sector 7	0.21%	0.09%	5
8	Sector 1	0.13%	0.05%	8

In Primary Care, we believe that there should be ongoing training in this pathology and Specialist-Primary care coordination, in order to raise awareness of the process, to enhance suspicion not only with the manifestation of classic and suggestive symptoms, but also in the case of less obvious ones such as hypertransaminasemia, certain types of dermatitis or hypercoagulability [25].

Prevalence differences among sectors underlines the need to establish diagnosis protocols, in order to raise awareness and enhance specialists’ training, so that they can give a clear and prompt diagnosis for CD before prescribing a gluten free diet for life. These measures lead not only to benefits for the patient, but also to important savings for the health system [26–28]. In this regard, it would be interesting to analyse the 1198 cases in the *weak evidence group* that were labelled under 729 additional descriptors related to the disease. Furthermore, it would be interesting to study their distribution according to health sector and specialist, as well as to see whether these cases are finally confirmed as CD or not, and how long it takes to reach a firm diagnosis.

Fasano [29] cites some of the causes that might be behind the CD underdiagnosis: [1] the serological markers are not always requested by general practitioners and pediatricians; [2] the small intestine biopsies are not carried out routinely when performing endoscopies; [3] problems when handling biopsy samples and [4] health insurance companies do not always cover the costs of these tests. Following ESPGHAN recommendations [3], serological markers and intestine biopsies should be included in an active and systematic search in cases of gastrointestinal symptomatology or other cases that are compatible with the disease, which is not a general practice in Aragón. In the case of point 2, this would increase the workload for pathological anatomy services and consumption in the endoscopy units. Nevertheless, this would probably be compensated by more and earlier diagnoses.

### Quality of the information

The fact that CD does not have its own ICPC code and the large number of associated descriptors used by general practitioners to refer to CD makes it difficult to reach a firm diagnosis and, therefore, any effective treatment. For example, the use of discouraged terms [1] such as *gluten intolerance* or *gluten-sensitive enteropathy* may mislead the diagnosis as they can easily be taken for a non-celiac gluten sensitivity instead of CD, especially when different general practitioners may share access to the same clinical record.

The lack of its own ICPC code and being coded under code D99 “Disease digestive system, other” in Primary Care lead family doctors to edit the descriptor in order to identify the diagnosis, creating confusion and unnecessary terminology variety. Therefore, the fact that this label is editable hinders diagnosis and epidemiological research. In 2011, this research team identified 2041 patients with CD in 93 descriptors using this same method. Hence, we can see that we are facing a growing problem, as we found 293 labels in 2016 that refer to the same disease. Over the last 25 years, this information tool has been introduced in 12 out of the 17 Spanish autonomous communities, as it is the best known and used information system in Spain, with a market share of 60% [30]. Consequently, we believe that these terminology difficulties can be found in other Autonomous Health Systems in Spain.

Additionally, the design of this information tool and label edition by family doctors allow the use of discouraged terms by the Oslo group to refer to CD (such as *gluten intolerance* or *enteropathy by gluten*), and its misuse prevents us from knowing how many patients are actually suffering from this disease.

Although CD may fulfill World Health Organization criteria for screening strategies in general population [31], it seems that there is no sufficient consensus on the relevance of promoting these programs [7, 13, 32–34]. While detection methods need to be improved, we believe that active case search strategies should be adopted. Thus, in line with the recommendations of the British Society of Gastroenterology [7] and the ESPGHAN [7], general practitioners should prescribe serological study in patients with mild gastrointestinal problems or with conditions associated to genetic risk, i.e., first-degree relatives, and an endoscopy with biopsy when the disease is suspected, e.g. malabsorption syndrome or a family history of CD. In patients with positive serology or symptomatology who are referred to endoscopy, duodenal biopsies are recommended. Finally, an HLA study for first-degree family could be considered as a way of discarding the disease, and thus avoiding future tests. These strategies have proved to be cost effective [28, 32, 34, 35].

Further investigation is necessary to search for the reasons behind prevalence differences, whether they are due to access to health resources, to sociodemographic characteristics, to different diagnosis criteria in the specialist teams or to differences in suspecting and detecting in Primary Care and a correct remittance to gastroenterological services.

In order to analyse this data, we must consider that, although the access to the Health System in Spain is free and universal, this data comes only from the Primary Care System and does not include those either from private care systems or the community.

The absence of a single diagnosis descriptor that covers CD means that we may be cautious when interpreting its prevalence estimations. In 2011 the implementation of electronic records in all Primary Care Centres of Aragon was concluded. Although we expected that a better knowledge of this tool would lead to a greater use and greater unanimous criteria when recording CD, 5 years later we can find that CD labelling has increased by 315%. Giving CD its own ICPC code will help to reduce the need to edit the description field and, therefore, facilitate diagnosis and research on this disease. We hope the protocol for the diagnosis of CD recently developed in Spain [36] helps to improve both case finding strategies and the use of unified correct terms.

Finally, this increase of detected CD cases and other chronic diseases in recent years, urges for a change in Spanish primary care health system. We believe that diagnosis and management of CD could be improved by implementing a new clinical governance framework in primary care [37]. All primary care systems should be patient centered and issues such as quality assurance, patient empowerment, patient and caregivers engagement, EBHC courses and the improvement of information and communication technologies will be of great help to improve early diagnosis of CD and adherence to the Gluten Free Diet in patients with CD in Spain.

## Conclusion

The prevalence of CD in Aragón is far from the general accepted figure of 1%. Additionally, the quality of the information available from Primary Care must be improved due to the number of different terms general practitioners use to refer to CD, as the use of many of them is discouraged nowadays.

## Data Availability

The datasets used and/or analysed during the current study are available from the corresponding author on reasonable request.

## Abbreviations

CD: Celiac disease
EHBC: Evidence- Based Health Care
ICPC D99: International Classification of Primary Care
WONCA: World Organization of National Colleges, Academies and Academic Associations of General Practitioners/Family Physicians

## References

[1] Ludvigsson JF, Leffler DA, Bai JC, Biagi F, Fasano A, Green PHR (2013). The Oslo definitions for coeliac disease and related terms. Gut..

[2] Comino I, Real A, Moreno L, Lorenzo L, Cornell H, Lopez-Casado M (2011). Is it the oat a toxic cereal for coeliac patients? Its depends on the variety. Ann Nutr Metab.

[3] Husby S, Koletzko S, Korponay-Szabó IR, Mearin ML, Phillips A, Shamir R (2012). European Society for Pediatric Gastroenterology, Hepatology, and Nutrition Guidelines for the Diagnosis of Coeliac Disease. J Pediatr Gastroenterol Nutr.

[4] Fasano A, Drago S, Thorpe M, Kryszak D, Fornaroli F, Horvath K (2003). Prevalence of Celiac disease in at-risk and not-at-risk groups in the United States: A large multicenter study. Arch Intern Med.

[5] Fasano A, Catassi C (2001). Current approaches to diagnosis and treatment of celiac disease: an evolving spectrum. Gastroenterology..

[6] Hadithi M (2007). Accuracy of serologic tests and HLA-DQ typing for diagnosing celiac disease. Ann Intern Med.

[7] Ludvigsson JF, Bai JC, Biagi F, Card TR, Ciacci C, Ciclitira PJ (2014). Diagnosis and management of adult coeliac disease: guidelines from the British Society of Gastroenterology. Gut..

[8] Rubio-Tapia A, Ludvigsson JF, Brantner TL, Murray JA, Everhart JE (2012). The prevalence of celiac disease in the United States. Am J Gastroenterol.

[9] Mustalahti K, Catassi C, Reunanen A, Fabiani E, Heier M, McMillan S (2010). The prevalence of celiac disease in Europe: results of a centralized, international mass screening project. Ann Med.

[10] Catassi C, Fabiani E, Rätsch IM, Coppa GV, Giorgi PL, Pierdomenico R (1996). The coeliac iceberg in Italy. A multicentre antigliadin antibodies screening for coeliac disease in school-age subjects. Acta Paediatr Suppl.

[11] Castaño L, Blarduni E, Ortiz L, Núñez J, Bilbao JR, Rica I (2004). Prospective population screening for celiac disease: high prevalence in the first 3 years of life. J Pediatr Gastroenterol Nutr.

[12] García Novo MD, Garfia C, Acuña Quirós MD, Asensio J, Zancada G, Barrio Gutierrez S (2007). Prevalencia de la enfermedad celiaca en donantes de sangre de la Comunidad de Madrid. Rev Esp Enfermedades Dig.

[13] Mariné M, Farre C, Alsina M, Vilar P, Cortijo M, Salas A (2011). The prevalence of coeliac disease is significantly higher in children compared with adults: changing prevalence of coeliac disease in Catalonia. Aliment Pharmacol Ther.

[14] Riestra S, Arranz E, Garrote JA (2011). Epidemiología de la enfermedad celíaca. Enfermedad celíaca: introducción al conocimiento actual de la enfermedad celíaca.

[15] Cilleruelo ML, Roman-Riechmann E, Sanchez-Valverde F, Donat E, Manuel-Ramos J, Martín-Orte E (2014). Spanish national registry of celiac disease: incidence and clinical presentation. J Pediatr Gastroenterol Nutr.

[16] Gujral N (2012). Celiac disease: prevalence, diagnosis, pathogenesis and treatment. World J Gastroenterol.

[17] Dixit R, Lebwohl B, Ludvigsson JF, Lewis SK, Rizkalla-Reilly N, Green PHR (2014). Celiac disease is diagnosed less frequently in young adult males. Dig Dis Sci.

[18] Llorente-Alonso MJ, Fernández-Aceñero MJ, Sebastián M. Gluten Intolerance: Sex- and Age-Related Features [Internet]. Canadian J Gastroenterol Hepatol. 2006; [cited 2018 Apr 20]. Available from: https://www.hindawi.com/journals/cjgh/2006/470273/abs/. 10.1155/2006/470273 PMC2660827 17111054

[19] Ivarsson A, Myléus A, Norström F, van der Pals M, Rosén A, Högberg L (2013). Prevalence of childhood celiac disease and changes in infant feeding. Pediatrics..

[20] Ravikumara M, Nootigattu V, Sandhu B (2007). Ninety Percent of Celiac Disease Is Being Missed. J Pediatr Gastroenterol Nutr.

[21] Fueyo Díaz R, Gascón Santos S, Magallón Botaya R. Caracterización de la población celíaca en Aragón: aspectos psicosociales de la adherencia a la dieta sin gluten. Zaragoza: Universidad de Zaragoza, Prensas de la Universidad; 2016.

[22] Lionetti E, Catassi C (2011). New clues in celiac disease epidemiology, pathogenesis, clinical manifestations, and treatment. Int Rev Immunol.

[23] Ivarsson A, Persson L, Juto P, Peltonen M, Suhr O, Hernell O (1999). High prevalence of undiagnosed coeliac disease in adults: a Swedish population-based study. J Intern Med.

[24] Virta LJ, Kaukinen K, Collin P (2009). Incidence and prevalence of diagnosed coeliac disease in Finland: results of effective case finding in adults. Scand J Gastroenterol.

[25] Lerner A, Blank M (2014). Hypercoagulability in celiac disease - an update. Autoimmun Rev.

[26] Chinburapa V, Green P, Edwards C, Gabinelle S (2004). Economic benefits of earlier diagnosis of celiac disease. Am J Gastroenterol.

[27] Green PHR, Neugut AI, Naiyer AJ, Edwards ZC, Gabinelle S, Chinburapa V (2008). Economic benefits of increased diagnosis of celiac disease in a national managed care population in the United States. J Insur Med N Y N.

[28] Hershcovici T, Leshno M, Goldin E, Shamir R, Israeli E. Cost effectiveness of mass screening for celiac disease is determined by time-delay to diagnosis and quality of life on a gluten free diet. Aliment Pharmacol Ther. 2010; [cited 2015 Aug 17]; Available from: http://doi.wiley.com/10.1111/j.1365-2036.2010.04242.x. 10.1111/j.1365-2036.2010.04242.x 20096017

[29] Fasano A (2005). Clinical presentation of celiac disease in the pediatric population. Gastroenterology..

[30] OMIAP - Software para atención primaria - stacks.es [Internet]. [cited 2015 Oct 21]. Available from: http://www.stacks.es/software-atencion-primaria

[31] Wilson JM, Jungner YG (1968). Principles and practice of mass screening for disease.

[32] Mearin ML, Ivarsson A, Dickey W (2005). Coeliac disease: is it time for mass screening?. Best Pract Res Clin Gastroenterol..

[33] Fasano A (2009). Should we screen for coeliac disease? Yes. BMJ.

[34] Mearin ML (2015). The prevention of coeliac disease. Best Pract Res Clin Gastroenterol.

[35] Shamir R, Hernell O, Leshno M (2006). Cost-effectiveness analysis of screening for celiac disease in the adult population. Med Decis Mak Int J Soc Med Decis Mak.

[36] Grupo de trabajo del Protocolo para el diagnóstico precoz de la enfermedad celíaca. Protocolo para el diagnóstico precoz de la enfermedad celíaca. [Internet]. Ministerio de Sanidad, Servicios Sociales e Igualdad. Servicio de Evaluación del Servicio Canario de la Salud (SESCS); 2018. Available from: https://www.mscbs.gob.es/profesionales/prestacionesSanitarias/publicaciones/DiagnosticoCeliaca.htm. [cited 2019 Jan 25].

[37] Buja A, Toffanin R, Claus M, Ricciardi W, Damiani G, Baldo V (2018). Developing a new clinical governance framework for chronic diseases in primary care: an umbrella review. BMJ Open.

